# Corticostriatal White Matter Integrity and Dopamine D1 Receptor Availability Predict Age Differences in Prefrontal Value Signaling during Reward Learning

**DOI:** 10.1093/cercor/bhaa104

**Published:** 2020-06-02

**Authors:** Lieke de Boer, Benjamín Garzón, Jan Axelsson, Katrine Riklund, Lars Nyberg, Lars Bäckman, Marc Guitart-Masip

**Affiliations:** 1 Neurobiology, Care Sciences and Society, Aging Research Center, Karolinska Institutet, Stockholm 171 65, Sweden; 2 Department of Radiation Sciences, Diagnostic Radiology, University Hospital, Umeå University, Umeå SE-901 87, Sweden; 3 Department of Integrative Medical Biology, Physiology, Umeå University, Umeå SE-901 87, Sweden; 4 Umeå Center for Functional Brain Imaging, Umeå University, Umeå 907 36, Sweden; 5 Max Planck UCL Centre for Computational Psychiatry and Ageing Research, University College London, London WC1B 5EH, UK

**Keywords:** aging, corticostriatal loops, dopamine, value-based decision making, white matter integrity

## Abstract

Probabilistic reward learning reflects the ability to adapt choices based on probabilistic feedback. The dopaminergically innervated corticostriatal circuit in the brain plays an important role in supporting successful probabilistic reward learning. Several components of the corticostriatal circuit deteriorate with age, as it does probabilistic reward learning. We showed previously that D1 receptor availability in NAcc predicts the strength of anticipatory value signaling in vmPFC, a neural correlate of probabilistic learning that is attenuated in older participants and predicts probabilistic reward learning performance. We investigated how white matter integrity in the pathway between nucleus accumbens (NAcc) and ventromedial prefrontal cortex (vmPFC) relates to the strength of anticipatory value signaling in vmPFC in younger and older participants. We found that in a sample of 22 old and 23 young participants, fractional anisotropy in the pathway between NAcc and vmPFC predicted the strength of value signaling in vmPFC independently from D1 receptor availability in NAcc. These findings provide tentative evidence that integrity in the dopaminergic and white matter pathways of corticostriatal circuitry supports the expression of value signaling in vmPFC which supports reward learning, however, the limited sample size calls for independent replication. These and future findings could add to the improved understanding of how corticostriatal integrity contributes to reward learning ability.

## Introduction

The ability to flexibly update one’s actions based on value-related changes in the environment deteriorates with age, as shown in decision-making studies comparing older and younger adults (Samanez-Larkin et al. 2012; Chowdhury et al. 2013b; Samanez-Larkin and Knutson 2015; de Boer et al. 2017). Animal studies and neuroanatomical evidence suggest that reward learning necessary for optimal value-based decision-making in changeable environments recruits corticostriatal loops (Haber and Knutson 2010; Seger et al. 2010; Haber 2016; Smittenaar et al. 2017). These loops are modulated by dopaminergic projections from the midbrain. Activity within the loop passing through the ventromedial portion of the striatum has consistently been associated with motivational aspects of behavior (Haber and Behrens 2014). Conversely, activity within the loops passing through the dorsolateral portion of the striatum is associated with converting cognitive and motivational signals into motor programs (Bornstein and Daw 2011).

The ventromedial prefrontal cortex (vmPFC) and nucleus accumbens (NAcc) are important nodes within the motivational portion of these loops. We have previously shown that value anticipation in vmPFC is related to performance on a two-armed bandit task (TAB) (de Boer et al. 2017). In that study, this signal proved weaker in a sample of older participants, compared with younger participants. Importantly, this value anticipation signal in vmPFC correlated with performance on the TAB, even when controlling for age. This suggested that as people age, the brain’s ability to produce a strong value signal needed to perform adaptive choices may change.

Aging affects the integrity of the dopaminergic system (Bäckman et al. 2000; Bäckman et al. 2010; Rieckmann et al. 2011) and white-matter tracts in the brain (Raz et al. 2005; Yang et al. 2016; Bennett et al. 2017). The deterioration of either or both of these systems could underlie worse adaptive value-based decision-making in older adults. We have already shown that dopamine (DA) D1-R availability in NAcc predicts the strength of the value signal in vmPFC (de Boer et al. 2017). Integrity of frontostriatal pathways as measured by diffusion weighted imaging (DWI) has previously proven the important of good performance in probabilistic reward learning tasks which measure value-based decision-making ability (Samanez-Larkin et al. 2012; van de Vijver et al. 2016). The relationship between these measures and value-based decision-making performance could stem from the fact that the prefrontal value signal necessary for making adaptive value-based choices cannot properly emerge if the dopaminergic and vmPFC-accumbens integrity are affected. This dual dependence on dopaminergic modulation in the NAcc and frontostriatal connectivity is supported by the recent observation that DA transporter binding potential in NAcc has an indirect effect on reinforcement learning behavior, through frontostriatal functional connectivity (Kaiser et al. 2018).

Based on this evidence, we hypothesized that vmPFC-accumbens white matter integrity would predict the strength of value anticipation signals in vmPFC. Given that D1-R availability in NAcc is also related to the strength of value anticipation in vmPFC in the data used in this study, we expected that one of these two measures could mediate the relationship of the other measure with value anticipation in vmPFC. Alternatively, both vmPFC-accumbens white matter integrity and D1-R availability in NAcc could independently predict the strength of value anticipation signals in vmPFC. We were also expecting to find a direct relationship between vmPFC-accumbens white matter integrity and behavioral performance on the TAB.

No study has previously investigated the combined effect of dopaminergic integrity and white-matter pathway integrity on probabilistic reward learning. Here, we test our hypotheses in a sample of 22 older and 23 young participants, whose data were part of a previously published study (de Boer et al. 2017). For these participants, we report previously unpublished DWI data, as well as functional MRI data during the TAB and DA D1-R availability data with positron emission tomography (PET) available to us (de Boer et al. 2017). We used a computational model to calculate subjective value for each participant on each trial (de Boer et al. 2017).

## Materials and Methods

### Participants

A total of 30 healthy, cognitively high functioning older adults aged 66–75 and 30 younger adults aged 19–32 were recruited through local newspaper advertisements in Umeå Sweden. The health of all potential participants was assessed before recruitment by a questionnaire administered via telephone by research nurses. The questionnaire enquired about past and present neurological or psychiatric conditions, head trauma, diabetes mellitus, arterial hypertension that required more than two medications, addiction to alcohol or other drugs, and bad eyesight. All participants were right-handed and provided written informed consent prior to commencing the study. Ethical approval was obtained from the Umeå Regional Ethical Review Board. Participants were paid 2000 SEK ($\sim$$225) for participation and earned up to 149 additional SEK ($\sim$$17) in the TAB.

In fMRI analyses, three older participants were excluded—one due to excessive head motion during fMRI scanning, one for only ever selecting one of the two stimuli in the task, and one due to a malfunctioning button box, resulting in no recorded responses. One additional older participant did not complete the full PET scan, but this participant’s fMRI and task data are still included in the analysis where possible. This resulted in a total of 57 participants for fMRI and task analysis (27 old [10 female], 30 young [18 female]) and 56 participants for PET analysis (26 old, 30 young). For DWI analysis, tracts between VS and vmPFC could not be reconstructed for 11 out of 57 participants. Thus, for DWI analysis, 46 participants were included (23 old [8 female], 23 young [14 female]). One of the older participants in this sample was the one that did not complete the PET scan, so for the full analysis, 45 participants (22 old, 23 young) were included.

All participants performed the Mini Mental State Examination (MMSE). Scores ranged from 26 to 30 in the young sample (mean = 29.40, SD = 0.97) and from 27 to 30 in the older sample (mean = 29.37, SD = 0.79), with no evidence of a difference between the two (*P* = 0.90). PET and fMRI scanning were planned 2 days apart. However, due to a technical problem with the PET scanner, 12 participants were scanned at a longer delay apart (range 4–44 days apart). On the MRI scanning day, participants completed the TAB and another unrelated task inside the MRI scanner. Participants also completed a battery of tasks outside the scanner. Only results from the TAB will be discussed here.

### Two-Armed Bandit Task

**Figure 1:**
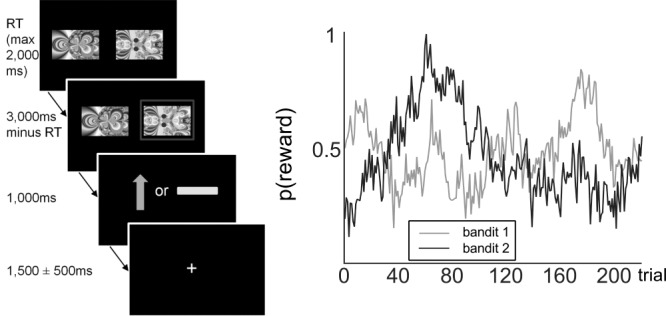
Left: schematic representation of a trial in the TAB. Participants were presented with two fractal images on each trial and selected one of them through a button press. The maximum response time was 2000 ms, meaning the trial would count as a miss if the response time exceeded this limit and the next trial would start immediately after the next intertrial interval. If one stimulus was selected, this option was highlighted with a red frame. After 1000 ms, participants were presented with the outcome: either a green arrow pointing upwards, indicating an obtained reward of 1 SEK ($0.11), or a yellow horizontal bar, indicating no win. Each image was randomly assigned a position on the screen (left or right) on each of the 2 × 110 trials of the experiment. Reward probabilities varied throughout the experiment. Right: varying reward probabilities for obtaining a reward for each bandit on the 220 trials of the experiment.

The TAB task was presented in Cogent 2000 (Wellcome Trust for Neuroimaging). Figure 1*a* depicts a schematic representation of one TAB trial. Participants were instructed to choose the fractal stimulus they thought to be most rewarding at each trial and were informed of the changing probability of obtaining a reward for each stimulus. These probabilities varied independently from one another. Probabilities were generated using a Gaussian random walk (Daw and Doya 2006). Before scanning, participants were presented with five practice trials. The same set of Gaussian random walks was used for all participants (Fig. 1), but the assignment of random walk to stimulus identity was counterbalanced across participants.

### Statistical Analysis of Brain and Behavior

All statistical analyses were performed using R version 3.5.3. As a measure of performance, we used the number of rewarded trials that each participant saw. This was equivalent to the amount of money each participant earned on the task (participants received 1 Swedish Crown per rewarded trial). Performance differences between groups were assessed with a one-tailed student’s *t*-test. We used the *lm* function in the R stats package to perform a number of multiple regressions that assessed the relationship between age, performance, white matter integrity, value anticipation in vmPFC, and DA D1-R availability in the NAcc. The assumptions of the regression models were checked by testing the residuals with Shapiro–Wilk’s test of normality and were considered normal at *P* > 0.05. Variance inflation factors (VIFs) were calculated with the *ols_vif_tol* function, part of the *olsrr* package. We considered predictor values to be overinflated if the VIF > 10 (O’brien 2007). In all of the analyses we performed, we included age as a covariate of no interest. However, we report all of the bivariate relationships without controlling for age in the multiple linear regression tables. As many of our predictors are collinear with age, controlling for age ensures that the observed relationships are robust across age groups. Thus, variables that are affected by age, but that can predict brain activity and performance beyond age, provide robust explanations for processes affected by age-related changes.

### Computational Analysis of Behavior

To calculate trial-by-trial choice values, we used a previously reported computational model, a variation on a Bayesian Observer that has been shown to outperform alternative models using standard model comparison methods (de Boer et al. 2017). For brevity, we will present only the winning model from this analysis. For model comparison statistics (including standard reinforcement learning models using the Rescorla–Wagner updating rule) and fitting procedures, we refer to our previous publication (de Boer et al. 2017).

The winning model uses a softmax decision rule, where action propensities (${m}_a(t)$) for each bandit were entered. A temperature parameter $\beta$ (with $\beta$ > 0) determined the probability that a participant chose each action a $\in${0,1} (corresponding to each bandit)

(1) \begin{equation*} P\left(a(t)=a\right)=\frac{\exp \left[\beta{m}_a(t)\right]}{\exp \left[\beta{m}_0(t)\right]+\exp \left[\beta{m}_1(t)\right]}, \end{equation*}

where ${m}_a(t)$ is the action propensity for bandit $a$ on trial $t$. In the winning model, the probability of obtaining a reward derived for each bandit was represented as a beta distribution (one for each bandit)

(2) \begin{equation*} {\theta}_a\sim \beta \left({\theta}_a;{\gamma}_a,{\varepsilon}_a\right), \end{equation*}

$\mathrm{where}\ {\theta}_a$ was updated upon observing an outcome of each trial. From these probability distributions, we derived the mean probability of getting a reward for each bandit and its variance. We will refer to the mean probability of obtaining a reward for each bandit on a given trial as the expected value ${Q}_a(t)$, which is calculated as follows:

(3) \begin{equation*} {Q}_a(t)=\frac{\gamma_a}{\left({\gamma}_a+{\varepsilon}_a\right)}. \end{equation*}

Additionally, the variance in reward probability, which was used to calculate the action propensities ${m}_a(t)$ (see further below), was defined as

(4) \begin{equation*} {V}_a(t)=\frac{\gamma_a{\varepsilon}_a}{{\left({\gamma}_a+{\varepsilon}_a\right)}^2\left({\gamma}_a+{\varepsilon}_a+1\right)}. \end{equation*}

The parameter values at $t=1$ in the beta distributions were 1 (${\gamma}_a(1)$ = ${\epsilon}_a(1)$ = 1). Therefore, ${Q}_0(1)={Q}_1(1)=0.5$, reflecting an expected value at chance level on the first trial, and ${V}_0(1)={V}_1(1)=0.143$, reflecting the maximum possible variance on the first trial, in line with a general uncertainty about the underlying reward probability distributions. Both parameters of each beta distribution are updated on each trial as follows: when bandit $a$ is chosen and a reward is obtained, ${\gamma}_a$ is increased by 1, ${\epsilon}_a$ is relaxed toward 1, and both ${\gamma}_{1-a}$ (1$-$*a* referring to the unchosen option) and ${\epsilon}_{1-a}$ are relaxed toward 1. Conversely, after reward omission, ${\epsilon}_a$ is increased by 1, but again, both ${\gamma}_{1-a}$ and ${\epsilon}_{1-a}$ are relaxed toward 1. Hence, for the chosen bandit,

$$ {\gamma}_{a(t)}\left(t+1\right)=\left(1-\omega \right){\gamma}_{a(t)}(t)+\omega +1;\mathrm{and} $$

(5) \begin{equation*} {\varepsilon}_{a(t)}\left(t+1\right)=\left(1-\omega \right){\varepsilon}_{a(t)}(t)+\omega;\quad if\ R(t)=1 \end{equation*}

$$ {\gamma}_{a(t)}\left(t+1\right)=\left(1-\omega \right){\gamma}_{a(t)}(t)+\omega; \mathrm{and} $$

(6) \begin{equation*} {\varepsilon}_{a(t)}\left(t+1\right)=\left(1-\omega \right){\varepsilon}_{a(t)}(t)+\omega +1;.\quad if\ R(t)=0 \end{equation*}

And for the unchosen bandit,

$$ {\gamma}_{1-a(t)}\left(t+1\right)=\left(1-\lambda \right){\gamma}_{1-a(t)}(t)+\lambda; \mathrm{and} $$

(7) \begin{equation*} {\varepsilon}_{1-a(t)}\left(t+1\right)=\left(1-\lambda \right){\varepsilon}_{1-a(t)}(t)+\lambda, \end{equation*}

where $\omega$ and $\lambda$ are separate individually fitted free parameters that determine the speed with which the reward probability distributions are updated (with $0<$$\omega$$<1$) and forgotten ($0<$$\lambda$$<1$).

In addition, the variance of the bandit that was not chosen on trail $t$ was added to the action propensity of that bandit on trial $t+1$. Hence, for the unchosen option,

(8) \begin{equation*} {m}_{1-a(t)}\left(t+1\right)={Q}_{1-a(t)}(t)+{\upsilon}^{\mathrm{unchosen}}{V}_{1-a}(t), \end{equation*}

where, if $\upsilon$ is positive, choices were favored if they had high variance and if they were not chosen on the previous trial, which can be interpreted as an exploration bonus.

Finally, a measure of confidence was added to the value of the bandit that was not chosen on trial $t$. Relative confidence was defined as the probability that a sample drawn from the distribution for bandit $a$ would be more likely to lead to a reward than a sample drawn from the distribution for bandit $1-a$. A relative confidence was added to the unchosen option at trial $t+1$

(9) \begin{equation*} {m}_{1-a(t)}\left(t+1\right)={Q}_{1-a(t)}(t)+{\upsilon}^{\mathrm{unchosen}}{V}_{1-a}(t)+\kappa{C}^{rel}(t), \end{equation*}

where $\kappa$ was an individually fitted parameter that weighted the relative confidence ${C}^{\mathrm{rel}}$ which was calculated as follows:

(10) \begin{equation*} {C}^{\mathrm{rel}}(t)=P\left({\theta}_{a(t)}>{\theta}_{1-a(t)}\right)-P\left({\theta}_{1-a(t)}>{\theta}_{a(t)}\right)=2P\left({\theta}_{a(t)}>{\theta}_{1-a(t)}\right)-1, \end{equation*}

where

(11) \begin{equation*} {C}_1(t)=P\left({\theta}_1>{\theta}_0\right)={\int}_{\theta_1=0}^1\mathrm{d}{\theta}_1\beta \left({\theta}_1;{\gamma}_1,{\varepsilon}_1\right){\int}_{\theta_0=0}^{\theta_1}\mathrm{d}{\theta}_0\beta \left({\theta}_0;{\gamma}_0,{\varepsilon}_0\right) \end{equation*}

and

(12) \begin{equation*} {C}_{1-a}(t)=1-{C}_a(t). \end{equation*}

### MRI Acquisition

Brain images were acquired on an MR750 3T scanner (GE Medical Systems), equipped with a 32-channel phased-array head coil. *T*_1_-weighted 3D-SPGR images were acquired using a single-echo sequence (voxel size: 0.5 × 0.5 × 1 mm, TE = 3.20, flip angle = 12°). DWI scans were acquired with a spin-EPI *T*_2_-weighted sequence (64 slices, voxel size = 1 × 1 × 2 mm, TR = 8000 ms, TE = 84.4 ms, FoV = 25 cm, flip angle = 90°), using three repetitions, with 32 independent directions (*b* = 1000 s/mm^2^) and six *b* = 0 images. Functional images were acquired using a *T*_2_*-sensitive gradient echo sequence (voxel size: 2 × 2 × 4 mm, TE = 30.0 ms, TR = 2000 ms, flip angle = 80°) and contained 37 slices of 3.4-mm thickness, with a 0.5-mm gap between slices. Volume acquisition occurred in an interleaved fashion. About 330 volumes were obtained for each of the two functional runs. During acquisition of fMRI time series, heart rate and respiratory data were collected using a breathing belt and a pulse oximeter.

### Functional MRI Analysis

In-house software (dicom2usb, http://dicom-port.com/) was used to de-identify all neuroimaging scans. Functional MRI analyses were performed in SPM8 (http://www.fil.ion.ucl.ac.uk/spm/software/spm8/). The preprocessing pipeline included slice-time correction, realignment, coregistration to the *T*_1_-weighted image, movement correction, and normalization to MNI space. For normalization, we used a diffeomorphic registration algorithm (DARTEL; Ashburner 2007) with spatial resolution after normalization 2 × 2 × 2 mm. Data were smoothed with a final Gaussian kernel equivalent to a standard 8 mm (see below). The fMRI time series data were high-pass filtered with a 128-s cut-off, and whitened with an AR(1) model. For each participant, the canonical hemodynamic response function was used to compute their statistical model.

The movement parameters produced by SPM’s coregistration algorithm showed that 15 participants moved >3 mm in any direction during functional runs. To correct for movement artifacts produced as a consequence of this, we used the ArtRepair toolbox (Mazaika et al. 2009; Levy and Wagner 2011). ArtRepair compares the amount of motion between volume acquisitions based on the mean intensity plot of all functional scans, and linearly interpolates scans in which motion exceeds a specified threshold. We used the recommended threshold value of 1.5% deviation from the mean intensity between scans. The average number of interpolated scans for our participants was 12.2 (1.8%) (SD = 19.6 [3.0%]), and one participant was excluded for showing movement >1.0 mm in >25% of scans, in line with ArtRepair’s recommendations. ArtRepair smooths the individual subject data with a Gaussian smoothing kernel of 4 mm before normalization and movement correction. A Gaussian kernel of 7 mm was then used for the normalization to MNI space, resulting in a smoothed, normalized image equivalent to a standard 8-mm smoothed normalized image.

We estimated a first-level general linear model (GLM) to look at activity corresponding to value anticipation in the brain. In this linear model, we parametrically modulated the time of choice by the expected value (*Q*) that belonged to the chosen option on each trial as calculated by the computational model described above. In addition, the outcome of each trial (whether the trial led to reward receipt or not) was included as a regressor at the time of outcome. This model included several other regressors of no interest to control for motion. These included SPM’s six motion regressors as well as 18 parameters that corrected for physiological noise, which we recorded with a heartbeat detector and a breathing belt during the scanning sessions. These regressors were calculated using the PhysiO toolbox version r671 (https://www.tnu.ethz.ch/en/software/tapas.html).

For each participant, we then calculated a contrast images by weighting the regressor of interest (*Q* at choice by 1). This contrast image was used at second level to perform a one-sample *t*-test across all participants. This resulted in a second-level map with a family-wise error (FWE) corrected threshold at *P* < 0.05, from which we extracted parameter estimates to be used in further analysis. For DWI analysis, the activity cluster in vmPFC at *P*(uncorrected) < 0.001 was used to facilitate the reconstruction of paths between NAcc and vmPFC.

### DWI Preprocessing and Analysis

Diffusion weighted scans were corrected for motion- and current-induced distortions with FSL’s eddy_correct. To further correct for geometric distortions, the images were nonlinearly aligned with the *T*_1_-weighted structural scan (Wu et al. 2008) with the ANTs software (Avants et al. 2011).

Tractograms were generated with the MRtrix software (Tournier et al. 2012) and filtered with the SIFT2 method (Smith et al. 2015), using anatomically constrained probabilistic tractography (Smith et al. 2012). We specified the two inclusion regions of interest (ROIs; vmPFC and NAcc, Supplementary Fig. 1) as binary mask images and accepted only streamlines that traversed both inclusion regions. We sampled until we recovered 100 streamlines between the vmPFC and NAcc ROIs. Subjects were excluded if >200 million streamlines were considered, but less than 20 were selected as probable (*n* = 11). FA maps were calculated using FSL’s dtifit. The tract formed by the reconstructed streamlines was used to mask the FA image, and the average within the tract became the individual’s measure of vmPFC-accumbens white matter integrity. FA values were thresholded at 0.2.

The inferior frontal fasciculus was selected as a control tract, as this tract is affected by aging (de Schotten et al. 2014), and could therefore serve as an appropriate control tract to ensure that relationships to FA and neural signals or performance were specific to the tract at hand. We also performed a control analysis including the inferior longitudinal fasciculus (Supplementary Table 1).

### PET Image Acquisition and Analysis

PET images were acquired on a 690 PET/CT scanner (GE Medical Systems). A low-dose helical CT scan (20 mA, 120 kV, 0.8 s/revolution) was used for PET attenuation correction. In order to minimize head movement during image acquisition, individually fitted thermoplastic masks were used to fixate the participants’ heads (Positocasts Thermoplastic; CIVCO medical solutions). PET scanning started after an intravenous bolus injection of 200 MBq of [^11^C]SCH23390. At the time of injection, a 55-min dynamic acquisition started (9 × 120 s, 3 × 180 s, 3 × 260 s, and 3 × 300 s), totaling 18 frames. Attenuation- and decay-corrected 256 × 256 pixel transaxial PET images were reconstructed to a 25-cm field-of-view using the Sharp IR algorithm (6 iterations, 25 subsets, 3.0-mm Gaussian post filter). Sharp IR is an advanced version of the Ordered Subset Expectation Maximization method for improving spatial resolution (Ross and Stearns 2010). The full-width half-maximum resolution was 3.2 mm. This protocol resulted in 47 tomographic slices per timeframe, with 0.98 × 0.98 × 3.3 mm^3^ voxels. Images were decay corrected to the start of the scan.

We used an ROI-based protocol to estimate nondisplaceable binding (BP_ND_). BP_ND_ values were obtained by coregistering the PET time series images to the *T*_1_-weighted MRI images using SPM. From the *T*_1_-weighted images, we segmented subcortical ROIs using the FIRST algorithm as implemented by FSL (Patenaude et al. 2011). Based on our previous publication (de Boer et al. 2017), we were interested in the NAcc. The cerebellum was segmented with the use of Freesurfer’s recon-all algorithm (Desikan et al. 2006) and used as a reference tissue due to the lack of DA D1 receptors in this structure (Hall et al. 1994). The average time activity curves were extracted across all voxels within each ROI. Then, BP_ND_ was calculated with the use of the Logan method (Logan et al. 1996) as implemented in imlook4d (imlook4d version 3.5, https://sites.google.com/site/imlook4d). BP_ND_ values were averaged across hemispheres for the NAcc.

It should be noted that [^11^C]SCH23390 does not only bind to D1-Rs in the brain—it also shows a (albeit much lower) affinity for 5-HT2A receptors (Ekelund et al. 2007; Slifstein et al. 2007). This has been shown to affect binding potentials in the cortex. In the NAcc, this is not as much of an issue, because the number of D1-Rs is many times greater than 5-HT2A receptors. In the cortex, however, 5-HT2A can represent up to 25% of the PET signal recorded with [^11^C]SCH23390 (Ekelund et al. 2007).

## Results

### Behavior

A total of 30 older (aged 66–75) and 30 younger (aged 19–32) participants performed a probabilistic reward learning while being scanned with fMRI. A schematic of the task as well as the variable reward probabilities for each bandit across the task are displayed in Figure 1. DWI pathways could only be reconstructed for 22 older and 23 younger participants. The behavioral results for the entire sample have been previously reported (de Boer et al. 2017). For completeness, we present here the behavioral results, both for the entire sample, and the DWI sample only.

**Figure 2:**
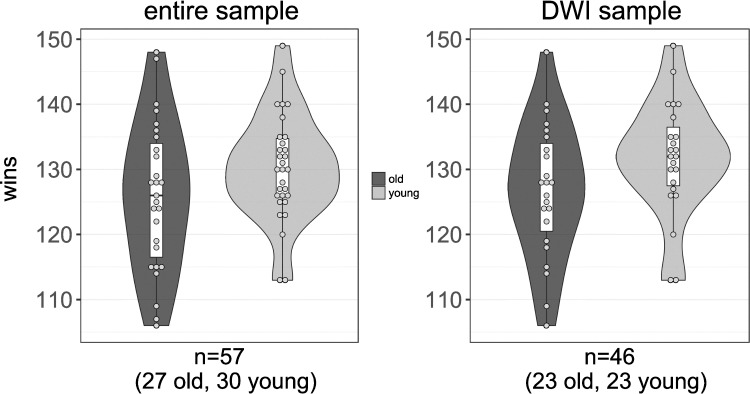
Behavioral performance on the TAB for older and younger participants, separately. Young participants performed marginally better than older participants, both in the whole sample (*P*[one-tailed] = 0.05, left figure, as previously published in de Boer et al. 2017), as well as in the DWI sample (*P*[one-tailed] = 0.04, right figure).

Participants earned between 106 and 149 Swedish Crowns on the task (11–16 USD, *M* = 128.21, SD = 10.00). Older participants performed slightly worse than younger participants in both the fMRI sample (*P*[one-tailed] = 0.05, Cohen’s *d* = 0.45, 95% confidence interval [CI]: −0.09 to 0.99) and the DWI subsample (*P*[one-tailed] = 0.04, Cohen’s *d* = 0.52, 95% CI: −0.08 to 1.13) (Fig. 2). A more elaborate comparison of performance between age groups is described in de Boer et al. 2017).

### Previous Findings in the DWI Subsample

Because the sample size in this study is limited compared with the previous publication (de Boer et al. 2017), we first confirmed that our previous findings held in the sample considered here. As in our previous study (de Boer et al. 2017), we used computational modeling to estimate the predicted expected value for each option as participants performed the TAB task (de Boer et al. (2017), see Materials and Methods). We used an GLM approach to look at fMRI activity corresponding to value anticipation in the brain (see Materials and Methods). In this linear model, we estimated the correlates of anticipated value by parametrically modulating the time of choice by the expected value (*Q*) that belonged to the chosen option on each trial. In addition, the outcome of each trial (whether the trial led to reward receipt or not) was included as a regressor at the time of outcome. The value signal in vmPFC is a reliable anticipatory signal thought to reflect value computation. We chose to focus on this signal, as it can more reliably be detected as a value signal in vmPFC than the expected value component of an RPE signal (Skvortsova et al. 2014).

From the first-level subject beta maps, we created a second-level map with an FWE-corrected threshold at *P* < 0.05. From this map, we used the vmPFC as a functional region of interest from which we extracted parameter estimates used in further analysis. The areas that were active during value anticipation are displayed in Table 1. We previously found that value-correlated anticipatory activity in vmPFC at the time of choice significantly correlated with the performance on the TAB task as indexed by the total amount of money won (de Boer et al. 2017). This relationship could also be observed in the subsample of our study for whom DWI data were of sufficient quality for tractography analysis [*r*(44) = 0.51, *P* < 0.001, 95% CI: 0.26–0.70]. This correlation survived correction for age [*r*(43) = 0.45, *P* = 0.002, 95% CI: 0.18–0.65] and was similar for the alternative measures of performance such as the percentage of optimal switches and the percentage of optimal choices (Supplementary Tables 2 and 3).

**Table 1:** Coordinates of clusters responsive to *Q* at the time of choice

Region	*x*	*y*	*z*	Cluster size	*z* score	*P*(FWE-corr, cluster)	*P*(FWE-corr, peak)
Left precuneus	−22	−52	12	1845	6.14	<0.001	<0.001
Right precuneus	12	−52	16	NA	5.62		<0.001
Right hippocampus	34	−36	−4	121	5.49	<0.001	0.001
vmPFC	−2	50	−8	187	5.44	<0.001	0.001
Right cuneus	12	−80	26	82	5.01	0.001	0.008

We also confirmed in the DWI sample that anticipatory value-related activity was correlated with DA D1-R BP_ND_ in NAcc. Thus, we correlated this anticipatory activity with D1-R BP_ND_ in NAcc in the entire sample, as well as in the DWI sample. Bivariate correlations were significant in the entire sample [*r*(54) = 0.41, *P* = 0.001, 95% CI: 0.17–0.61], as well as in the DWI sample [*r*(43) = 0.40, *P* = 0.006, Table 4]. When entered into a multiple regression with age, D1_R BP_ND_ in NAcc was the only significant predictor of the value signal in vmPFC in the entire sample (*P* = 0.04, 95% CI of standardized beta weight: 0.02–0.83, for age: 95% CI: −0.39 to 0.41). This predictor was, however, not significant in the DWI sample only (*P* = 0.10, 95% CI of standardized beta weight: −0.07 to 0.83, for age: 95% CI: −0.48 to 0.42).

### Relationship Between White-Matter Integrity, Behavior, D1 BP_ND_, and Neural Correlates of Value Anticipation

**Figure 3:**
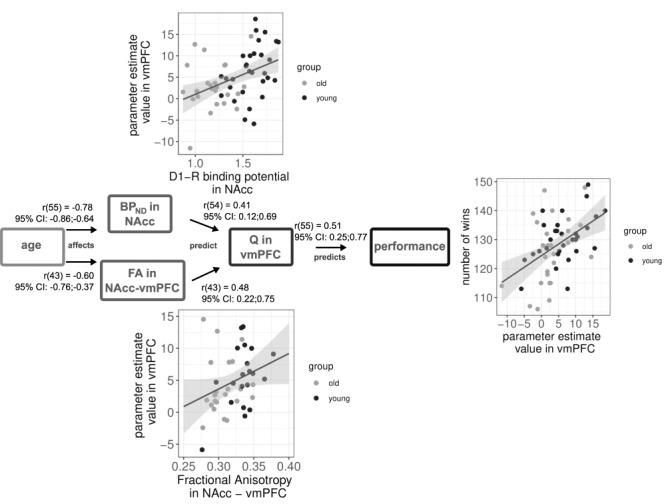
Observed relationships (bivariate correlations) between variables investigated in this study. Age is related to both a lower D1-R BP_ND_, and lower FA in the connection between vmPFC and NAcc. Both of these variables predict the expected-value signal in vmPFC during choice. This expected-value signal proved important for performance on value-based decision-making. Numbers and CIs on the lines indicate bivariate correlations and their 95% CI. The multiple regression coefficients for these relationships can be found in Tables 2 and 4. Note that in a multiple regression, D1-R BP_ND_ in NAcc did not survive as a significant predictor, possibly due to a lack of statistical power in the reduced sample, or as the result of a type 1 error.

Next, we tested the hypothesis that vmPFC-accumbens white matter integrity was correlated to the anticipatory value signal in vmPFC. We performed tractography analysis to reconstruct the pathway between the NAcc ROI, which was used to obtain BP_ND_ estimates, and the vmPFC ROI in which we saw value anticipatory activity. We used fractional anisotropy (FA) in this pathway as the measure of interest, in line with previous work (Samanez-Larkin et al. (2012)). White-matter integrity in this pathway was significantly different between younger and older participants [*M*_young_ (SD) = 0.34 (0.03), *M*_old_ (SD) = 0.31 (0.02), *P* < 0.001, Cohen’s *d* = 1.43]. We correlated these FA values to the value-anticipatory activity in vmPFC. This correlation proved significant [*r*(44) = 0.48, *P* = 0.001, 95% CI: 0.22–0.68, Fig. 3]. When entered in a multiple regression, only FA, not age, was a significant predictor of value-anticipatory activity in vmPFC and survived correction for age (*P* = 0.01, 95% CI of standardized beta weight: 0.12–0.79, for age: 95% CI: −0.39 to 0.28).

We next investigated our hypotheses combining all predictors of anticipated value in vmPFC. We tested the hypothesis that FA in the vmPFC-accumbens tract would mediate the relationship between D1-R availability in NAcc and the strength of the value anticipation signal in vmPFC. Alternatively, we predicted that FA in the vmPFC-accumbens tract and D1-R availability in NAcc could independently predict the strength of value anticipation signals in vmPFC. To test these hypotheses, we first correlated FA in the vmPFC-accumbens tract with D1-R availability in NAcc. In line with a full mediation hypothesis, these variables would have to be correlated, and their correlation would be expected to cancel out the relationship between D1 receptor availability and the strength of the expected value signal in the vmPFC. However, these variables were not significantly correlated (*r* = 0.14, *P* = 0.40). Thus, we observed no evidence supporting a mediatory relationship between FA in the vmPFC-accumbens tract and D1-R availability in the NAcc.

In order to test the hypothesis that the two could independently predict the strength of the expected value signal in the vmPFC, we performed a multiple linear regression to investigate how D1-R BP_ND_ and FA in the vmPFC-accumbens pathway were related to the anticipated value signal in vmPFC. The results of this multiple regression analysis are displayed in Table 2. Our result was in contrast with the hypothesis that predicted a mediation of the relationship between D1-R BP_ND_ in NAcc and anticipatory value signal in vmPFC by vmPFC-accumbens FA. Instead, it supported the hypothesis that both BP_ND_ in NAcc and vmPFC-accumbens FA were independent predictors of the anticipated value signal in vmPFC (Table 2; ${\beta}_{\mathrm{age}}$=0.30, *P* = 0.211, ${\beta}_{\mathrm{D}1-\mathrm{R}}$ = 0.41, *P* = 0.052, ${\beta}_{\mathrm{FA}}$ = 0.49, *P* = 0.006, Table 2). It should be pointed out that the significance of D1-R BP_ND_ in NAcc as a predictor was at what is sometimes referred to as trend level. Given the previously observed significant relationship between these variables, and the reduced sample size in this study, we choose to present this relationship as one contributing significant variance, rather than being nonsignificant. If we assumed that the effect size of the relationship between D1-R in NAcc and Q in vmPFC is at the small-to-medium effect size of 0.3 as we reported in de Boer et al. (2017)), we had significantly reduced power in the subsample reported here to detect that relationship. However, the limited sample size of this analysis should also caution against the over interpretation of this effect: we cannot exclude the possibility that this relationship is a type 1 error. The addition of another predictor of white matter integrity did not change the significance of these predictors (inferior frontal fasciculus, Supplementary Table 1), suggesting that this relationship is specific to the white matter pathway that we investigated here. The model with both FA and D1-R BP_ND_ in NAcc proved superior in predicting the expected-value signal in vmPFC compared with a model with FA or D1-R BP_ND_ as a single predictor beyond age (Table 3).

**Table 2:** Univariate and multivariate standardized coefficients (95% CIs) predicting the expected-value signal in vmPFC

Dependent: *Q* in vmPFC	Coefficient (univariate)	Coefficient (multivariate)
Age	−0.32 (−0.61 to −0.03, *P* = 0.030)	0.30 (−0.17 to 0.76, *P* = 0.211)
D1-R in NAcc	0.41 (0.12 to 0.69, *P* = 0.006)	0.41 (−0.00 to 0.82, *P* = 0.052)
vmPFC-accumbens FA	0.48 (0.22 to 0.75, *P* = 0.001)	0.49 (0.15 to 0.83, *P* = 0.006)

Both D1-R BP_ND_ and FA in the connection between NAcc and vmPFC independently predicted the strength of this value signal. It should be noted that we present these effects as independent here, based on the previously reported significant relationship between BP_ND_ in NAcc and the expected-value signal in vmPFC. However, this relationship exceeded the threshold for significance when entered into the regression with age and FA in NAcc-vmPFC. Univariate coefficients represent the result of bivariate correlations, whereas multivariate coefficients represent multiple regression coefficients of a model including all predictors.

**Table 3:** Model comparison demonstrating that a model predicting value anticipation in vmPFC with both D1-R in NAcc and vmPFC-accumbens FA is superior to a model with one of the predictors only

Model	BIC	Adjusted *R*-squared
Age + D1	135	0.122
Age + FA	131	0.203
Age + D1 + FA	130	0.256

Finally, given previous findings by Samanez-Larkin et al. (2012), we wanted to understand how vmPFC-accumbens white matter integrity influenced the relationship between the anticipatory value signal in vmPFC and performance on the TAB task. Therefore, we investigated the relationship between all of these variables and performance on the TAB task. Table 4 shows the univariate and multivariate estimates of the relationships between value anticipation in vmPFC, D1-R BP_ND_ in NAcc, and the amount of money won on the task. Univariate coefficients represent the result of bivariate correlations, whereas multivariate coefficients represent multiple regression coefficients of a model including all predictors. The table also shows the VIF for each predictor variable. VIF is a measure of how inflated the value of a predictor is in a model as a result of multicollinearity between the predictors. A rule of thumb states that a VIF > 10 is cause for further investigation (O’brien 2007). However, in our model, no VIF exceed 3.3 (Table 4). In a multiple regression, only the anticipatory value signal in vmPFC proved the predictive of performance on the TAB (Table 4; ${\beta}_{\mathrm{age}}$ = −0.33, *P* = 0.176; ${\beta}_{\mathrm{D}1-\mathrm{R}}$ = −0.20, *P* = 0.373; ${\beta}_{\mathrm{FA}}$ = −0.11, *P* = 0.550; ${\beta}_{\mathrm{Q}-\mathrm{vmFPC}}$ = 0.54, *P* = 0.002). Other measures reported in de Boer et al. (2017)), such as the proportion of optimal choices and optimal switch behavior, showed similar relationships to these neural predictors, with *Q* in vmPFC as the only significant predictor (Supplementary Tables 2 and 3). This is in contrast with our hypothesis, which predicted that we would observe a direct relationship between vmPFC-accumbens FA and behavioral performance. In Figure 3, we summarize the observed relationships.

**Table 4:** Univariate and multivariate standardized coefficients (95% CIs) predicting the number of wins on the TAB from D1-R BPND in NAcc, FA in the connection between NAcc and vmPFC, and the expected-value signal in vmPFC

Dependent: wins	Coefficient (univariate)	Coefficient (multivariate)	VIF
Age	−0.29 (−0.58 to 0.00, *P* = 0.053)	−0.33 (−0.82 to 0.16, *P* = 0.176)	3.268340548
D1-R in NAcc	0.23 (−0.08 to 0.53, *P* = 0.138)	−0.20 (−0.64 to 0.24, *P* = 0.373)	2.670655726
vmPFC-accumbens FA	0.26 (−0.03 to 0.55, *P* = 0.081)	−0.11 (−0.49 to 0.27, *P* = 0.550)	1.973339025
*Q* in vmPFC	0.51 (0.25–0.77, *P* < 0.001)	0.54 (0.22–0.86, *P* = 0.002)	1.442440738

The expected-value signal is the only significant predictor of behavior in a multiple regression model. The VIF is a measure of how inflated the variance of each predictor is due to multicollinearity of the predictors. VIFs in this model are below 10, which is considered within acceptable range. Univariate coefficients represent the result of bivariate correlations whereas multivariate coefficients represent multiple regression coefficients of a model including all predictors.

## Discussion

We showed in a sample of 23 young and 22 older participants that the strength of a anticipatory value signal in vmPFC is predicted by: 1) DA D1-R BP_ND_ in NAcc and 2) vmPFC-accumbens white matter integrity. The anticipatory value signal in vmPFC is an important predictor of good performance on the probabilistic reward learning task used in this study. Although DA D1-R BP_ND_ in NAcc and vmPFC-accumbens white matter integrity did not directly predict the performance on the task, these new results suggest that both measures of corticostriatal integrity are crucial for the emergence of the value anticipatory signal.

Our findings are in line with previous studies showing that frontostriatal white matter integrity is important for value-based decision-making. Specifically, one study by Samanez-Larkin et al. (2012) showed that the integrity of white matter on the pathway between NAcc and medial PFC could predict the performance on a probabilistic monetary incentive learning task. This relationship between performance and white matter integrity survived correction for age. Similarly, a study by van de Vijver et al. (2016) has shown that some, but not all parameters reflecting integrity in frontostriatal white matter, were related to the measures of probabilistic reward learning. Frontostriatal white matter integrity was also found to be predictive of the development of delay of gratification in a longitudinal study with adolescents (Achterberg et al. 2016), suggesting that good decision-making and frontostriatal white matter integrity go hand in hand.

We previously reported that DA D1-R availability in NAcc was a significant predictor for the anticipatory value signal in vmPFC. DA receptors in the NAcc have often been implicated in successful probabilistic reward-learning (Koch et al. 2000; Salamone and Correa 2012; Shiner et al. 2012; Chowdhury et al. 2013a). It is believed that DA neurons report reward prediction errors (Schultz et al. 1997; Day et al. 2007) to target structures such as the NAcc. These dopaminergic signals in NAcc appear to be a crucial hallmark of learning (Pessiglione et al. 2006; Jocham et al. 2011) and are also necessary to continue making good decisions based on learned values (Shiner et al. 2012; Collins and Frank 2014).

A limitation of this study is that we could not reconstruct white-matter pathways in 11 out of 57 participants and, thus, lost a considerable amount of power in detecting relationships between DA, FA, and *Q* in vmPFC. Despite this, we could replicate the previously observed relationship between performance and value anticipation in vmPFC in the DWI subsample (de Boer et al. 2017). The two predictors we found could each explain variance in the multiple regression predicting activity in vmPFC, although the previously observed relationship between DA D1-R availability and activity in vmPFC proved fragile once the sample size was reduced. We believe that this may be related to our inability to detect this relationship with small-to-medium effect size in a small sample like the one used here. However, we cannot exclude the possibility of this relationship constituting a type 1 error. This relationship should, therefore, be replicated in an independent sample to ensure that this is not a false positive result. If this relationship does exist, this suggests that both high dopaminergic integrity in NAcc as well as high integrity in relevant white-matter pathways, contribute to a strong value signal and subsequently good performance. The fact that DA D1-R availability and vmPFC-accumbens white matter integrity predict the strength of the value anticipation signal in vmPFC is in line with the well-established theory that decision-making and reward learning are dependent on corticostriatal loops (Seger 2009; Haber 2016). Activity in these loops is modulated by dopaminergic signals that project from the midbrain to the striatum (Haber and Knutson 2010). This dopaminergic modulation allows for the emergence of value signals in prefrontal cortex. Computational evidence suggests that striatal D1 receptors, specifically, play an important role in this iterative gating process (Gruber et al. 2006).

Although we did not observe any direct relationship between frontostriatal white matter integrity and behavioral performance, our results suggest an indirect relationship. Frontostriatal white matter integrity predicted value anticipation in the vmPFC which in turn predicted behavioral performance on the TAB task. Value anticipation signals in vmPFC have consistently been shown to be crucial for the ability to perform probabilistic reward learning tasks (Noonan et al. 2010; Bartra et al. 2013; Halfmann et al. 2016), as it is the most flexible brain region when it comes to quick value computation (Haber 2016), with computations occurring just before or during an action. Our results suggest that the age-related attenuation in the anticipatory value signal may be in part attributed to the decreased integrity of the frontostriatal tract associated with older age. The fact that we did not observe a direct relationship between frontostriatal white matter integrity and behavioral performance may stem from the design of our task. Whereas previous studies have used tasks with stationary probabilistic contingencies (Samanez-Larkin et al. 2012, 2014; Eppinger et al. 2013), the reward probabilities for each stimulus fluctuated according to a random walk. Whereas our task design may maximize the occurrence of prediction errors during probabilistic reward learning, it also promotes the exploration of unchosen options which may additionally depend on frontal mechanisms unrelated to the frontostriatal path that we studied here (Boorman et al. 2009, 2011).

The exploration involved in this task may also provide an explanation for the marginal performance difference between older and younger participants. Some previous studies have shown that older adults perform on average somewhat, but not dramatically, worse than younger participants on value-based decision-making tasks (Samanez-Larkin et al. 2008, 2014; Lighthall et al. 2018). This difference is usually larger when the task involves probabilistic decisions that require participants to learn to update behavior based on changing reward contingencies (Mell et al. 2005; Mata et al. 2011; Worthy and Maddox 2012), as compared with decision-making tasks where learning is not required. Here, we only found marginal differences on a probabilistic learning task. This lack of behavioral difference in our and other studies is mirrored by a lack of difference between these older and younger participants in the strength of neural signals reflecting reward prediction errors in the NAcc (de Boer et al. 2017; Lighthall et al. 2018). Differential exploration between the two age groups and the relatively small sample size may provide an explanation for a relatively behavioral difference between age groups. Additionally, the older adults in this study are cognitively high functioning, with MMSE scores above 26.

We present an important contribution to the mechanistic understanding of decision-making in probabilistic environments. Despite the fact that our sample size is small for evaluating a mediation with five variables, our observations demonstrate two separate predictors of the integrity of corticostriatal circuitry, which may both contribute to the emergence of strong anticipatory value signals important for successful decision-making. These findings, taken together, provide insights into how age-related decay in the integrity of frontostriatal white matter as well as dopamine D1 receptor availability in the NAcc may underlie an impairment in probabilistic reward learning performance in some older adults.

## Supplementary Material

suppfig1_bhaa104

suppfig2_bhaa104

extended_data_bhaa104

## Data Availability

The code and processed behavioral and neural data used to create the figures in this paper are available at https://github.com/liekelotte/DWI.
