# Supplementary figures and images for: Corticostriatal White Matter Integrity and Dopamine D1 Receptor Availability Predict Age Differences in Prefrontal Value Signaling during Reward Learning
Source: Cereb Cortex. 2020 Jun 2;30(10):5270–80. doi: 10.1093/cercor/bhaa104 (PMC7472214; doi:10.1093/cercor/bhaa104)

vmPFC

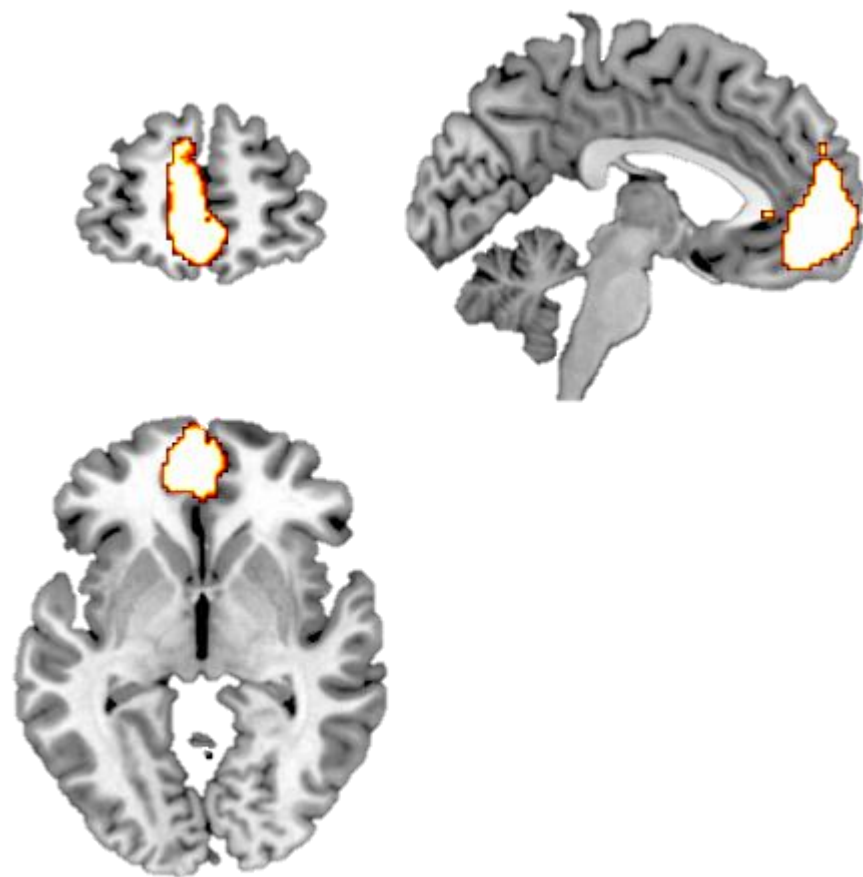

-4, 55, 3

NAcc

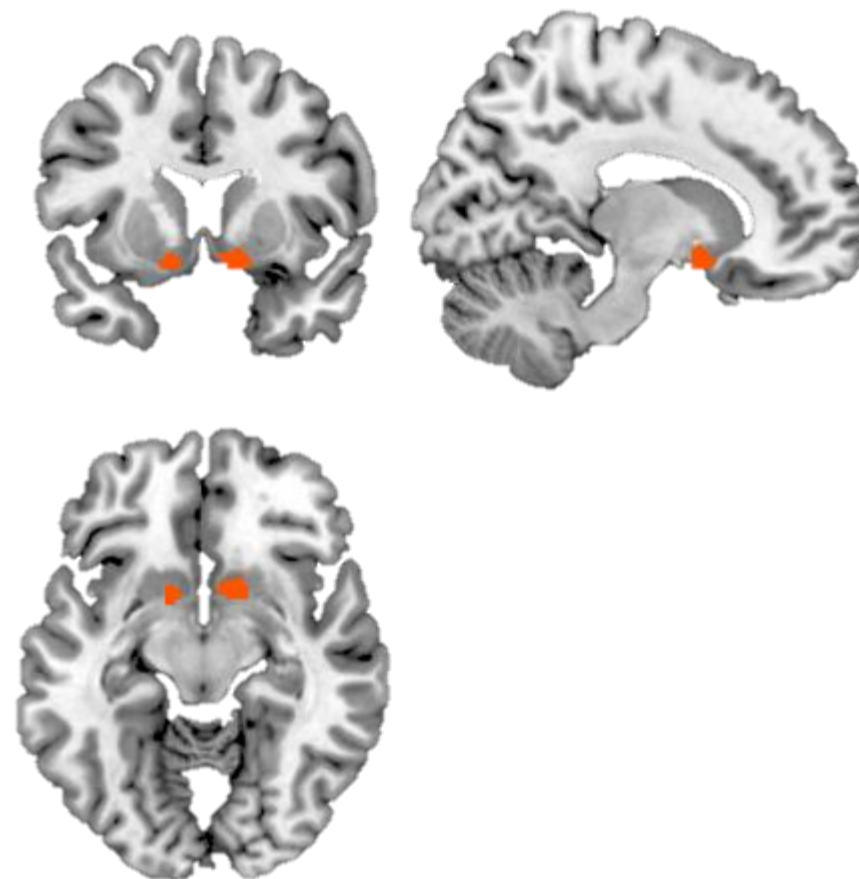

12, 10, -10

Supplement: suppfig1_bhaa104 [file suppfig1_bhaa104.pdf]

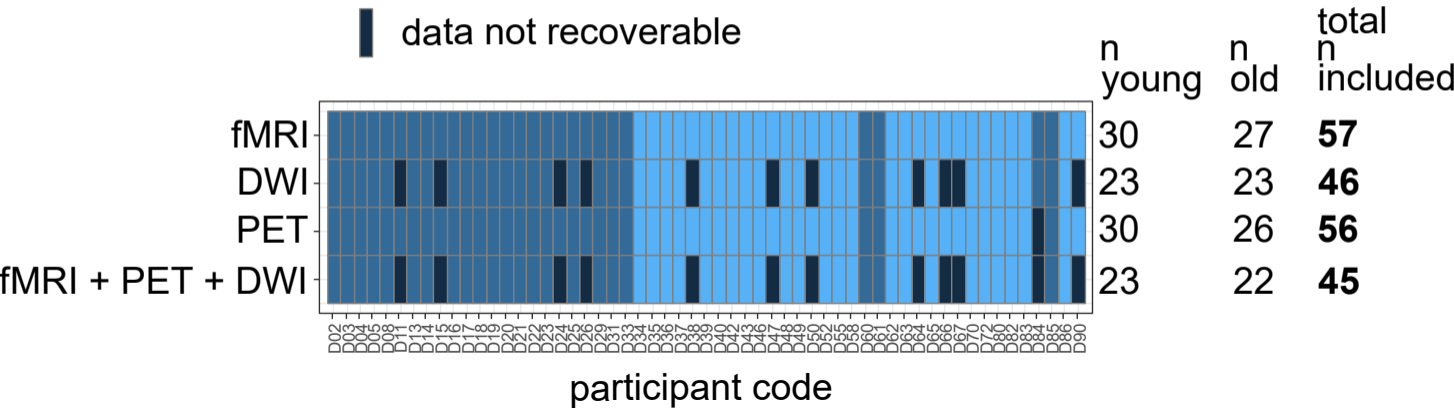

Supplement: suppfig2_bhaa104 [file suppfig2_bhaa104.pdf]
