# Supplementary material for: Corticostriatal White Matter Integrity and Dopamine D1 Receptor Availability Predict Age Differences in Prefrontal Value Signaling during Reward Learning
Source: Cereb Cortex. 2020 Jun 2;30(10):5270–80. doi: 10.1093/cercor/bhaa104 (PMC7472214; doi:10.1093/cercor/bhaa104)
Supplement: extended_data_bhaa104 [file extended_data_bhaa104.pdf]

# Supplementary tables for: Corticostriatal white matter integrity and dopamine D1 receptor availability independently predict age differences in prefrontal value signaling during reward learning.

Supplementary table 1. Univariate and multivariate standardized coefficients (95% confidence intervals) predicting the expected-value signal in vmPFC. Both D1-R $BP_{ND}$ and FA in the connection between NAcc and vmPFC independently predicted the strength of this value signal.

| Dependent: Q_vmPFC | Coefficient (univariate) | Coefficient (multivariate) |
|---|---|---|
| age | -0.32 (-0.61 to -0.03, p=0.030) | 0.25 (-0.23 to 0.73, p=0.296) |
| D1-R in NAcc | 0.41 (0.12 to 0.69, p=0.006) | 0.41 (-0.01 to 0.83, p=0.058) |
| accumbofrontal FA | 0.47 (0.20 to 0.74, p=0.001) | 0.42 (0.04 to 0.81, p=0.032) |
| inferior frontal fasciculus FA | -0.23 (-0.52 to 0.07, p=0.130) | -0.08 (-0.38 to 0.23, p=0.612) |

Supplementary table 2 and 3. Univariate and multivariate standardized coefficients (95% confidence intervals) predicting the proportion of optimal switches and optimal choices on the TAB from D1-R $BP_{ND}$ in NAcc, FA in the connection between NAcc and vmPFC, and the expected-value signal in vmPFC. The expected-value signal is the only significant predictor of behavior in a multiple regression model. Univariate coefficients represent the result of bivariate correlations, whereas multivariate coefficients represent multiple regression coefficients.

| Dependent: % optimal switches | Coefficient (univariate) | Coefficient (multivariate) |
|---|---|---|
| age | -0.20 (-0.50 to 0.10, p=0.184) | -0.00 (-0.51 to 0.51, p=0.999) |
| D1-R in NAcc | 0.19 (-0.12 to 0.49, p=0.221) | -0.03 (-0.50 to 0.44, p=0.892) |
| vmPFC-accumbens FA | 0.30 (0.01 to 0.59, p=0.046) | 0.15 (-0.24 to 0.55, p=0.443) |
| Q in vmPFC | 0.43 (0.15 to 0.70, p=0.003) | 0.37 (0.03 to 0.71, p=0.034) |

| Dependent: % optimal choices | Coefficient (univariate) | Coefficient (multivariate) |
|---|---|---|
| age | -0.30 (-0.59 to -0.01, p=0.046) | -0.50 (-1.02 to 0.01, p=0.054) |
| D1-R in NAcc | 0.13 (-0.17 to 0.44, p=0.389) | -0.36 (-0.82 to 0.11, p=0.131) |
| vmPFC-accumbens FA | 0.20 (-0.10 to 0.50, p=0.186) | -0.11 (-0.50 to 0.29, p=0.582) |
| Q in vmPFC | 0.34 (0.05 to 0.62, p=0.021) | 0.37 (0.03 to 0.71, p=0.033) |
